# Nurses’ knowledge of hypertension care and associated factors in the Northern Region of Ghana

**DOI:** 10.4102/hsag.v31i0.3307

**Published:** 2026-05-13

**Authors:** Sylvia P. Adzitey, Furaha Akimanimpaye, Talitha Crowley

**Affiliations:** 1 School of Nursing, Faculty of Community and Health Sciences, University of the Western Cape, Cape Town, South Africa; 2 Tamale Teaching Hospital, Tamale, Ghana

**Keywords:** hypertension, hypertension care, Ghana, knowledge, nurses

## Abstract

**Background:**

Hypertension is the main risk factor for cardiovascular-related morbidities and mortalities. Nurses play a pivotal role in hypertension care, encompassing screening, detection, management, patient education and self-management counselling.

**Aim:**

This study assessed nurses’ knowledge of hypertension care and associated factors in the Northern Region of Ghana.

**Setting:**

Tamale Metropolis of the Northern Region of Ghana.

**Methods:**

A descriptive cross-sectional study was conducted among nurses. Using a census approach, 410 eligible nurses working in hypertension care across the study hospitals were invited to participate. Of these, 266 consented and completed a self-reported validated questionnaire. The IBM Statistical Package for the Social Sciences programme version 30 was used to process the data and perform descriptive and inferential statistics.

**Results:**

The mean hypertension care knowledge score was 51.47% ± 11.76%. About 4% had good knowledge, 57.1% average knowledge, and 38.7% had poor knowledge. While nurses demonstrated good knowledge of accurate blood pressure measurement (82.4%), their knowledge of hypertension diagnosis (35.1%) and management (48.8%) was poor. The following were significantly associated with overall knowledge of hypertension care: hospital of practice (*p* = 0.021), age (*p* = 0.007), nursing category (*p* = 0.002), qualification (*p* = 0.001), years of practice in the present unit (*p* = 0.021) and hypertension in-service training (*p* = 0.027).

**Conclusion:**

This study revealed significant gaps in nurses’ knowledge of hypertension care and highlighted the need for continuous professional development to bridge knowledge gaps and sustain clinical competence.

**Contribution:**

The study provided evidence on nurses’ knowledge of hypertension care in Ghana, which can inform the development of tailored interventions to improve management and patient outcomes.

## Introduction

On a global scale, the leading cause of non-communicable disease-related mortalities is cardiovascular diseases (ed. World Health Organization [WHO] 2023), with hypertension being the main risk factor (Passi-Solar et al. 2020). High-income countries are experiencing a decreasing prevalence of hypertension, with many having well-controlled blood pressure (BP) readings; however, low- and middle-income countries (LMICs) are witnessing an increase in both prevalence rates and uncontrolled hypertension (Al-Rousan et al. 2020; WHO 2023). Hypertension is a chronic, persistent, and lifelong condition requiring strategies to effectively manage it (Samson et al. 2024). One in three adults worldwide is affected by hypertension, yet nearly half remain unaware of their condition, and only one in five achieves well-controlled BP (WHO 2025). The WHO defines hypertension as a systolic blood pressure (SBP) ≥ 140 mmHg, a diastolic blood pressure (DBP) ≥ 90 mmHg or the use of antihypertensive medication (WHO 2023). Sustained BP control, < 140/80 mmHg, is crucial to prevent cardiovascular morbidity and mortality; controlled BP saves lives (Brunström & Carlberg 2018; WHO 2021).

Nurses play a central role in the prevention and management of hypertension. Their knowledge, competencies and practices influence early detection, adherence to treatment and the promotion of patient self-management (International Council of Nurses 2019; WHO 2013). In many LMICs, including Ghana, nurses are often the first point of contact for individuals with hypertension, which places them in a pivotal position to support patients in managing this chronic condition (Alrasheedi et al. 2025; Gyamfi et al. 2017). Effective self-management support empowers patients with knowledge, skills and resources (Ryan & Sawin 2009). Therefore, nurses must be adequately knowledgeable about hypertension care, including accurate blood pressure measurement (ABPM), diagnostic criteria, risk factor identification, patient education and the management of hypertension (Himmelfarb, Commodore-Mensah & Hill 2016; Rivera-Figueroa 2023; Younis et al. 2024).

A study in Mongolia revealed limited knowledge, practice and confidence of nurses regarding hypertension, although they had a desirable attitude (Myanganbayar et al. 2019). Addressing the challenges of hypertension requires that nurses, as frontline providers, be clinically competent and equipped to provide education and self-management support (Hannan et al. 2022; Himmelfarb et al. 2016; Younis et al. 2024).

In Ghana, the prevalence of hypertension has risen steadily in recent decades, with increasing contributions to cardiovascular and stroke-related morbidity and mortality (Sanuade, Boatemaa & Kushitor 2018). Despite the availability of effective medicines, poor BP control remains a challenge (Champaneria, Patel & Oroszi 2023). Moreover, community-based studies in LMICs have reported inadequate knowledge, attitudes and practices regarding hypertension among both patients and the general public (Ahmed, Maphasha & Okeke 2025; Chimberengwa, Naidoo & Group 2019). These gaps perpetuate poor control rates despite treatment availability.

Existing hypertension research in Ghana has predominantly focused on hypertension prevalence, risk factors and clinical outcomes (Atibila et al. 2021; Bosu & Bosu 2021; Sanuade et al. 2018; Sumaila, Asumah & Dassah 2021). Despite nurses’ role in hypertension care, limited studies in Ghana, particularly in the Northern Region, have examined their knowledge and competence in hypertension care (Adler et al. 2020; Gyamfi et al. 2017). This gap constrains efforts to design effective interventions that can improve patient outcomes. This study, conducted as part of a broader project to develop hypertension self-management support guidelines for nurses, aimed to assess nurses’ knowledge of hypertension care and associated factors in the Northern Region of Ghana.

## Research methods and design

### Study design and setting

This study employed a quantitative descriptive cross-sectional design to examine nurses’ knowledge of hypertension care and associated factors. The study was conducted in the Tamale Metropolis, the capital of the Northern Region of Ghana. Specifically, it was done at the three major government hospitals, namely Tamale Teaching Hospital (TTH), Tamale Central Hospital (now the Northern Regional Hospital) (TCH) and Tamale West Hospital (TWH). Tamale Teaching Hospital is a tertiary-level healthcare facility, and the other two provide secondary care. These hospitals were selected because they serve as the main referral centres in the Northern Region. Also, because hypertension and other chronic diseases management in Ghana are mainly anchored in secondary level and above hospitals.

### Study population and sampling

The study population comprised nurses working in the outpatient department (OPD), hypertension clinic and the medical wards of the selected hospitals. Newly qualified rotation and newly employed nurses who have worked for less than 6 months were excluded. The estimated average population from which the sample was drawn was approximately 410 nurses (TTH: 230 [56%], TCH: 90 [22%] and TWH: 90 [22%]). The sample size was initially calculated using a precision-based method, based on a population size of 410 nurses, a 95% confidence interval and a 5% margin of error, yielding in an estimated sample of 199 respondents. Respondents were proportionally allocated across hospitals. However, rather than limiting recruitment to this number, the census approach was adopted. To maximise response rate and enhance representativeness, all eligible nurses across the participating hospitals were invited to participate.

### Study instrument

The study instrument was adapted from two validated instruments (Campbell et al. 2017; Serrat-Costa et al. 2022). The original instrument of Campbell et al. (2017) has 16 knowledge questions (with question 4 having six sub-questions and question 10 having two sub-questions, making a total of 22 questions), while Serrat-Costa et al. (2016) has 23 questions. These two instruments were integrated, and some questions were removed for contextual reasons, resulting in 34 knowledge questions. The 34-item questions assessed knowledge under three domains: ABPM (Section A), hypertension diagnosis (Section B) and hypertension management (medication and lifestyle) (Section C). The questions included multiple choice questions (including choosing all that apply; yes, no and I don’t know) and short answers.

### Reliability and validity

Before using the survey instrument, Campbell et al. (2017) indicated that the survey’s specific questions need to be examined for their local and national applicability. In the instrument of Serrat-Costa et al. (2022), a Cronbach’s α greater than 0.7 was obtained. In this study, a reliability test was done for the individual domains, resulting in a Cronbach’s alpha of 0.51 for the BP measurement domain. The hypertension diagnosis and hypertension management domains resulted in Cronbach’s alphas of 0.49 and 0.51, respectively. However, when all three domains were computed, the overall reliability analysis for the instrument yielded a Cronbach’s alpha of 0.66, indicating a moderate internal consistency. Although it is slightly below the recommended threshold, this value could be attributed to the high heterogeneity of the content (Izah, Sylva & Hait 2023; Tavakol & Dennick 2011).

Campbell et al. (2017) involved hypertension and primary care experts to determine the face validity of the knowledge, attitude and practice (KAP) instrument; it was also pilot-tested among primary healthcare professionals (sample size not stated). The same instrument was used in two studies (Myanganbayar et al. 2018, 2019). The second instrument (Serrat-Costa et al. 2022) also met all Rasch-Item Response Theory (Rasch-IRT) model criteria: item- and person-fit measurement, unidimensionality, local independence, invariance, targeting and reliability. Face validity is defined as the degree to which test respondents view the content of a test and its items as relevant to the context in which the test is being administered (Allen, Robson & Iliescu 2023), while content validity refers to the relevance, comprehensiveness and comprehensibility of the content of an instrument (Mokkink et al. 2025). In this present study, content validity was examined by involving subject experts (a cardiologist and a senior nurse in hypertension care) to assess the appropriateness and relevance of the test and items as they appear to the respondent. They recommended that some questions were outside the scope of nursing practice and should be removed. The two co-authors (also nurses) thoroughly reviewed the questionnaires to determine their relevance to the test purpose. A pilot test was conducted to assess item ambiguity, ease of answering and applicability to this study as a means of determining the face validity (Allen et al. 2023). Because the adapted instruments are existing validated instruments, they were examined to determine their applicability in the Ghanaian context. The pilot test resulted in the removal of five questions from the instrument because most respondents complained about the ambiguity of those questions.

### Data collection

The Principal Investigator (PI, SPA) engaged with the nurse managers of the respective study sites to introduce the study to the eligible nurses. The PI (SPA) is a staff member of the TTH and was therefore able to approach the eligible nurses. Although the principal PI is a staff member of TTH, she does not hold any managerial role over the eligible nurses. Formal permission to engage nurses was obtained from the nurse managers of the various units, who acted as the institutional gatekeepers. Eligible nurses were informed about the study either through unit managers or the PI and research assistant, and participation was entirely voluntary. The research team approached the identified nurses at the close of their work shift to prevent interfering with their working hours. They were informed about the purpose of the study, and participant information sheets were provided for them to review for details and clarity. Upon agreeing to participate, they were given consent forms to sign. Paper-based questionnaires were self-administered and returned anonymously by dropping them into a box in the nurse managers’ office, which was retrieved daily by the PI to minimise any perceived coercion and to ensure confidentiality. Other respondents who were interested in learning more about the study chose to hand in the completed questionnaires to the PI and ask questions. Email addresses and personal identifiers were not collected through the online version of the questionnaires. Most respondents answered paper-based questionnaires, and some responded online via REDCap (Harris et al. 2009), which was shared with respondents via WhatsApp. Paper-based responses were later entered into REDCap by the PI and a research assistant; at every 10th entry of the questionnaire into REDCap, the PI and research assistant (RA) cross-checked to ensure accurate data entry. Ten responses were excluded for not meeting the inclusion criteria: (1) students and (2) newly qualified with less than 6 months of working experience in hypertension care; they were thus not entered into REDCap.

### Data analysis

Data from REDCap were exported to the Statistical Package for the Social Sciences (SPSS) version 30 for analysis (IBM Corporation 2024). During data cleaning, four more responses were eliminated because of incomplete data. Results are presented using descriptive and inferential statistics. The continuous variables (age, years of practice experience and number of years in the present unit) were summarised as mean ± s.d. Categorical variables (gender, hospital name, ward type, nursing category, level of education, hypertension training and knowledge questions) were calculated as frequencies and percentages. Knowledge scores were first divided into three sections and calculated as the number of correct or desirable answers divided by the number of items in each section (8 items in section A and 13 items in sections B and C) surveyed and expressed as a percentage. The total knowledge score was the total number of correct or desirable responses divided by the total number of items surveyed (34 items) and multiplied by 100%. Knowledge level was further categorised as good, average and poor. A score of ≥ 75% meant good knowledge, a score of 50% to ˂ 75% represented average knowledge and poor knowledge was a score < 50%. This categorisation was done to help distinguish between high and moderate knowledge levels.

In the study of Myanganbayar et al. (2019), a score of ˂ 50% represented low knowledge and ≥ 50% represented good knowledge. The authors of the instrument indicated that there is no strict scoring when using the instrument; authors adopting the instrument should contextualise it (Campbell et al. 2017). The Chi-square test was used to test for associations between demographic variables and knowledge categories. A significance level of 0.05 was applied.

### Ethical considerations

This study, part of a broader project, received ethics approval from the Biomedical Health Research Ethics Committee (BMREC) of the University of the Western Cape, dated 05/12/2024 (BM24/10/6), and the Ghana Health Service Ethics Review Committee (GHS-ERC), dated 26/05/2025 (GHS-ERC:01103/35). Upon receiving ethics approval, written permission was obtained from the study sites. Written informed consent was obtained from all respondents involved in the study. Participants were assured of anonymity and confidentiality; they were not required to share any personal identifiers. They were also assured that their participation was voluntary and they could decline from the study without explaining to the researchers their reason for not continuing.

## Results

### Demographic data

A total of 266 (64.9% response rate) nurses working in the outpatient and medical inpatient units from three different hospitals participated in the study. The mean age was 33.16 ± 4.62 years; the ages were categorised into two: that is, 23–33 years and 34+, with 56.4% below 34 years. The mean years of experience was 7.04 ± 4.36 years, and years of practice in their present units were 3.23 ± 1.96 years.

Table 1 presents the categorical demographic data of respondents; the majority of the nurses were from the TTH, representing 61.7% (*n* = 164). Nurses were asked to indicate the type of ward they work in their respective hospitals; the responses included OPD, Male or Female Medical ward, Hypertension Specialist Clinic, TTH Polyclinic OPD and ward. These were categorised into inpatient and outpatient units; 200 (75.2%) work in inpatient wards, and the rest in outpatient units. Fifty-three per cent (*n* = 141) of respondents were females. The majority of the respondents are Registered General Nurses (Professional nurses) (81.2%, *n* = 216), and the rest are enrolled (16.2%, *n* = 43) and Nurse Specialists (2.6%, *n* = 7). Just over half of the respondents reported a Bachelor of Nursing qualification (50.4%, *n* = 134), 28.9% (*n* = 77) a Diploma in Nursing, 14.4% (*n* = 41) a certificate and a few (5.3%, *n* = 14) a postgraduate degree. Respondents were asked if they received any training on hypertension prevention and management, and over half (56.8%, *n* = 151) indicated they received no hypertension training.

**TABLE 1:** Demographic data of respondents (*N* = 266).

Variable	Frequency	%
**Hospital name**		
Tamale Teaching Hospital	164	61.7
Tamale Central Hospital	48	18.0
Tamale West Hospital	54	20.3
**Ward type**		
Inpatient	200	75.2
Outpatient	66	24.8
**Gender**		
Male	123	46.2
Female	141	53.0
Prefer not to say	2	0.8
**Nursing category**		
Nurse specialist	7	2.6
Registered general nurse	216	81.2
Enrolled nurse	43	16.2
**Level of education**		
Certificate	41	15.4
Diploma	77	28.9
Bachelors	134	50.4
Postgraduate	14	5.3
**Hypertension training**		
Received no training	151	56.8
Training within the last 2 years	73	27.4
Training for more than 2 years	42	15.8

### Knowledge of accurate blood pressure measurement

**FIGURE 1:**
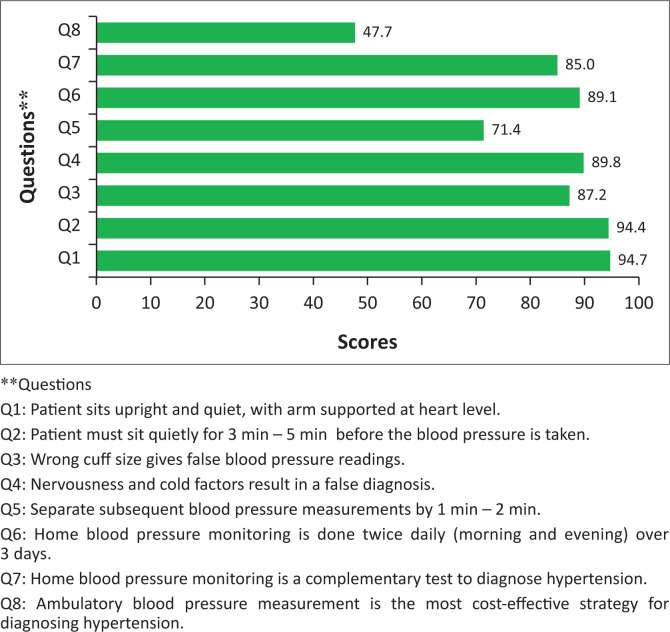
Knowledge of accurate and continuous blood pressure measurement (*N* = 266).

Respondents’ mean knowledge score for ABPM was 82.42% ± 16.65%, indicating that they were well informed in this category (Table 2). Respondents knew that the patient must sit upright and quiet, with the arm supported at heart level (94.7%, *n* = 252) and that patients must sit quietly for 3 min – 5 min before BP is taken (94.4%, *n* = 251). Further, 87.2% (*n* = 232) knew that the wrong cuff size gives a false reading. However, only 47% (*n* = 127) knew that the use of ambulatory BP measurement is a cost-effective strategy for diagnosing hypertension (Figure 1).

**TABLE 2:** Knowledge domains (*N* = 266).

Variable	*N*	Minimum	Maximum	Mean	s.d.
Knowledge score on accurate blood pressure measurement (A)	266	0.00	100.00	82.42	16.65
Knowledge score on diagnosis (B)	266	0.00	92.31	35.05	15.74
Knowledge score on medication and lifestyle management (C)	266	7.69	92.31	48.84	16.26

**Total knowledge score (A + B + C)**	266	14.71	91.18	51.47	11.76

s.d., standard deviation.

### Knowledge of hypertension diagnosis

**FIGURE 2:**
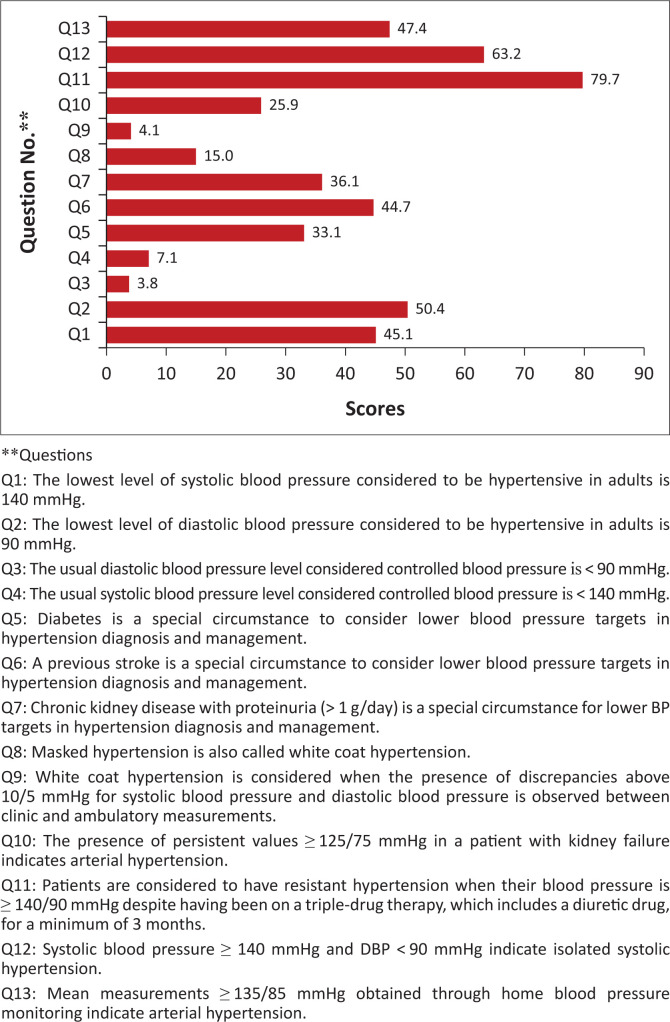
Knowledge of hypertension diagnosis (*N* = 266).

Respondents’ knowledge of the diagnosis of hypertension was poor, with a mean of 35.05 ± 15.74 (Table 2). Figure 2 presents details of respondents’ understanding of hypertension diagnosis. Under the individual variables, 79.7% (*n* = 212) were well informed about what resistant hypertension means. About 45% (*n* = 120) of respondents knew the lowest level of SBP considered hypertensive in adults, and 50.4% (*n* = 134) knew the lowest level of DBP considered hypertensive. Their understanding of BP values in controlled BP was poor, 3.8% (*n* = 10) and 7.1% (*n* = 19) for SBP and DBP, respectively.

Their knowledge of masked hypertension and white coat hypertension (which is potentially caused by inaccurate home and clinic BP measurements, respectively) was also low, scoring 15% and 4.1%, respectively.

### Knowledge of medication and lifestyle management of hypertension

**FIGURE 3:**
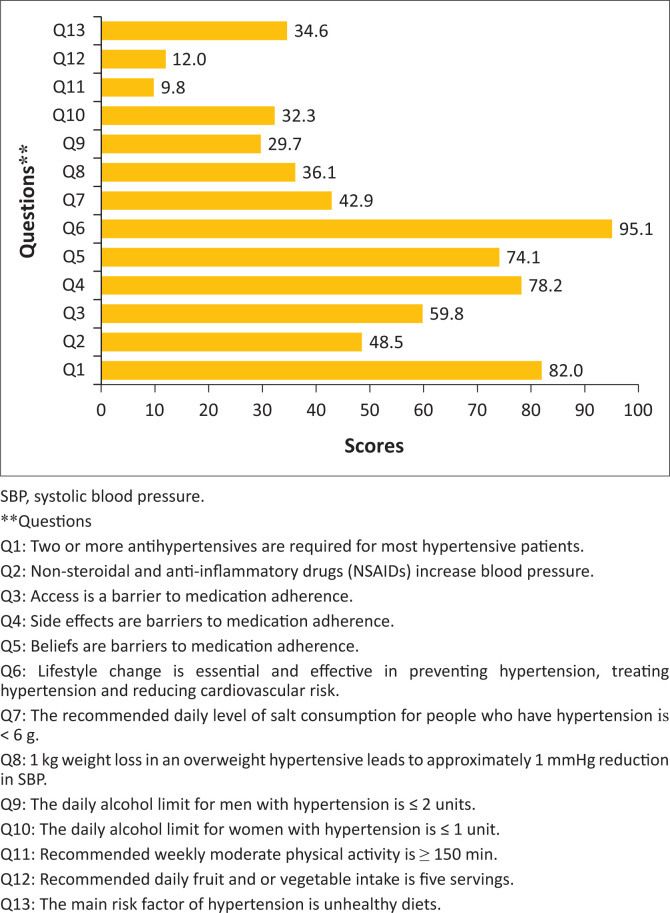
Knowledge of medication and lifestyle management of hypertension (*N* = 266).

The third category was medication and lifestyle management (Figure 3). The mean knowledge score was 48.84% ± 16.26% (Table 2). Most respondents knew that lifestyle change is essential and effective in managing hypertension and preventing cardiovascular events (95.0%, *n* = 253). Respondents were also well informed that two or more antihypertensives are required by most hypertensive patients (82.0%, *n* = 218), and 78.2% (*n* = 208) knew that side effects are one of the barriers to medication adherence. Regarding lifestyle, however, nurses’ knowledge was low; only 42.9% (*n* = 114) knew that the recommended daily level of salt consumption for people who have hypertension is < 6 g. A few respondents were well informed that 1 kg weight loss in an overweight hypertension patient leads to approximately 1 mmHg reduction in SBP. Regarding the alcohol intake limit for hypertension patients, 29.7% (*n* = 79) and 32.3% (*n* = 86) were well informed that male patients should limit to ≤ 2 and ≤ 1 for female patients, respectively. With the recommended weekly physical activity and fruits and vegetables intake, 9.8% (*n* = 26) and 12.0% (*n* = 32), respectively, were well informed.

### Overall hypertension knowledge

**FIGURE 4:**
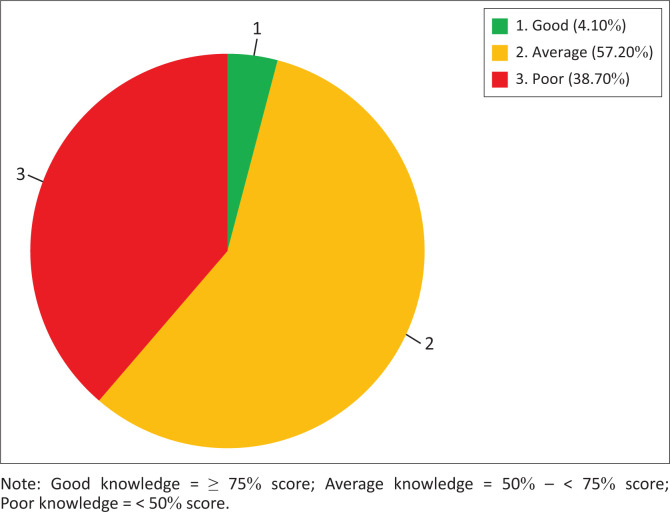
Nurses’ knowledge level of hypertension (*N* = 266).

The overall knowledge score of respondents was 51.47% ± 11.76%. Nurses’ overall knowledge level was categorised into three: good knowledge, for respondents who had ≥ 75%, average knowledge, for those who had 50% to < 75% and poor knowledge, for scores less than 50%. Figure 4 presents nurses’ total knowledge level; 4.1% (*n* = 11) had good knowledge, 57.1% (*n* = 152) had average knowledge and 38.7% (*n* = 103) had poor knowledge of hypertension care.

Respondents’ overall knowledge of hypertension care was further categorised under domains for ABPM (Figure 1), diagnosis (Figure 2) and medication and lifestyle management (Figure 3).

### Association of knowledge level with demographic variables

Table 3 shows the association between nurses’ knowledge of hypertension care and selected demographic variables. Regarding hospital of practice, more than half of the nurses with good knowledge were from TWH (54.5%, *n* = 6). In the average knowledge group, the largest proportion was from TTH (65.8%, *n* = 100) and TCH (19.1%, *n* = 29). Among those with poor knowledge, the majority were from TTH (58.3%, *n* = 60). A statistically significant association was found between hospital and knowledge level (χ^2^ = 11.508, degrees of freedom [*df*] = 4, *p* = 0.021)

**TABLE 3:** Association of knowledge level with demographic variables (*N* = 266).

Variable	Knowledge level						χ^2^	*df*	*p*-value
	Good (*n* = 11)		Average (*n* = 152)		Poor (*n* = 103)				
	*n*	%	*n*	%	*n*	%
**Hospital**							11.508	4	**0.021**
Tamale Teaching Hospital	4	36.4	100	65.8	60	58.3	-	-	-
Tamale Central Hospital	1	9.1	29	19.1	18	17.5	-	-	-
Tamale West Hospital	6	54.5	23	15.1	25	24.3	-	-	-
**Inpatient or outpatient unit**							3.041	2	0.219
Inpatient	7	63.6	110	72.4	83	80.6	-	-	-
OPD	4	36.4	42	27.6	20	19.4	-	-	-
**Gender**							1.016	4	0.907
Male	5	45.5	74	48.7	44	42.7	-	-	-
Female	6	54.5	77	50.7	58	56.3	-	-	-
**Prefer not to say**	-	-	1	0.7	1	1.0	-	-	-
**Age range (years)**							9.926	2	**0.007**
Age 23–33	4	36.4	76	50.0	70	68.0	-	-	-
Age 34+	7	63.6	76	50.0	33	32.0	-	-	-
**Nursing category**							17.266	4	**0.002**
Nurse specialist	1	9.1	4	2.6	2	1.9	-	-	-
Registered general nurse	7	63.6	135	88.8	74	71.8	-	-	-
Enrolled nurse	3	27.3	13	8.6	27	26.2	-	-	-
**Highest qualification**							17.661	2	**< 0.001**
Below bachelors	5	45.5	51	33.6	62	60.2	-	-	-
Bachelor’s and above	6	54.5	101	66.4	41	39.8	-	-	-
**Years of nursing practice**							7.692	4	0.104
1–5	2	18.2	65	42.8	55	53.4	-	-	-
6–10	5	45.5	45	29.6	30	29.1	-	-	-
11+	4	36.4	42	27.6	18	17.5	-	-	-
**Years in present ward**							7.755	2	**0.021**
1–5	7	63.6	126	82.9	94	91.1	-	-	-
6+	4	36.4	26	17.1	9	8.7	-	-	-
**Hypertension training after school**							7.245	2	**0.027**
Received no training	6	54.5	76	50.0	69	67.0	-	-	-
Had training within the past 1–5 or more years	5	45.5	76	50.0	34	33.0	-	-	-

Note: Bold values indicates statistical significance.

OPD, outpatient department; *df*, degrees of freedom.

Regarding the unit of practice, among nurses with average knowledge, most were in the inpatient unit (72.4%, *n* = 110) and a smaller proportion in the outpatient unit (27.6%, *n* = 42). Poor knowledge was more common among nurses in the inpatient unit (80.6%, *n* = 83) than in the outpatient unit (19.4%, *n* = 20). Good knowledge was observed in both units, with 63.6% (*n* = 7) in the inpatient unit and 36.4% (*n* = 4) in the outpatient unit. There was no statistically significant association between unit and knowledge level (χ^2^ = 3.041, *df* = 2, *p* = 0.219). Good knowledge was divided as 45.5% and 54.5% between males and females, with no statistical differences (χ^2^ = 11.16, *df* = 4, *p* = 0.907). Age categories demonstrated a significant difference (χ^2^ = 9.926, *df* = 2, *p* = 0.007), with poor knowledge observed more in nurses aged 23–33 years, 68.0% (*n* = 70). The nursing category showed a significant association with knowledge (χ^2^ = 17.266, *df* = 4, *p* = 0.002), with Registered General Nurses making up the largest share of the average knowledge group (88.8%) and the good knowledge group (63.6%). Although Nurse Specialists were few, they were distributed across all categories. Among nurses with good knowledge, 9.1% (*n* = 1) was a nurse specialist, and among average knowledge and poor knowledge groups, 2.6% (*n* = 4) and 1.9 (*n* = 2), respectively, were Nurse Specialists. Educational qualification was significantly associated with knowledge (*p* < 0.001). Good knowledge (54.5%) and average knowledge (66.4%) was observed more in nurses with a bachelor’s degree or higher, while 60.2% of nurses with qualifications below a bachelor’s had poor knowledge. Although years of practice were not significantly associated with knowledge (*p* = 0.104), the data showed that nurses with 1–5 years of experience were more likely to have poor knowledge (53.4%), while those with 6–10 years demonstrated good knowledge (45.5%). The number of years in the present ward was significantly associated with knowledge (*p* = 0.021). Among nurses with average knowledge, most had 1–5 years in their present ward (82.9%, *n* = 126), and among those with poor knowledge, the majority also had 1–5 years in the ward (91.1%, *n* = 94). Conversely, nurses with longer ward experience (6+ years) accounted for a higher proportion of those with good knowledge (36.4%, *n* = 4).

Finally, in-service hypertension care training was significantly associated with knowledge (*p* = 0.027). Among those with good knowledge, nearly half had received training (45.5%, *n* = 5). In the average knowledge group, half had received training (50.0%, *n* = 76). By contrast, in the poor knowledge group, most had not received training (67.0%, *n* = 69).

## Discussion

This study assessed nurses’ knowledge of hypertension care at the three major public hospitals in the Northern Region as part of a broader research project to develop self-management support guidelines for nurses in Ghana.

The findings revealed that 53.0% of the nurses were females, which is lower than the 77.0% and 98.0% reported in similar studies in other LMICs (Akter & Suhag 2020; Begum et al. 2023). The mean age was 33.16 ± 4.62 years, with the youngest nurse aged 23 and the oldest 50.

Significant gaps in respondents’ knowledge of different categories of hypertension care were revealed.

Respondents’ theoretical knowledge of ABPM was good. This score could be explained by the fact that BP measurement is a daily and more frequent activity, as indicated in their high scores in items related to routine BP measurement in hospital settings. A contrasting finding was reported in a study specifically on BP measurement in a national cardiac centre in Karachi, Pakistan. It was reported that the theoretical knowledge (96.0%) and skills (74.7%) of BP measurement among study nurses were poor (Younis et al. 2024). In Girona, Spain, a study by Serrat-Costa et al. (2022) concluded that ‘primary care nurses in the studied region do not have sufficient theoretical knowledge to detect hypertension’. Suboptimal theoretical knowledge of BP measurement was also reported among nurses and doctors in Nigeria; 89.1% (*n* = 115/129) of nurses had poor knowledge (Ojo et al. 2018).

Diagnosing hypertension begins with accurate BP measurement and the BP readings (Muntner et al. 2019; Serrat-Costa et al. 2016). Respondents were asked about cut-off SBP and DBP values for diagnosing hypertension, clinic and home BP measurements (which can lead to white-coat and masked hypertension) and ambulatory BP measurement. The presence of other comorbidities like diabetes, a previous stroke and kidney failure could also affect the threshold of BP values in diagnosing hypertension (Serrat-Costa et al. 2022). Nurses need to understand these phenomena as vital members of the team-based care to make a meaningful contribution to patient management. Nurses’ role in diagnosing hypertension is crucial, as they lead screening, detection and ongoing education for patients and communities (Himmelfarb et al. 2016). In this study, respondents’ knowledge of hypertension diagnosis was poor. This reveals a gap in hypertension care in the healthcare system, where, anecdotally, it is believed that doctors are the ones responsible for making a diagnosis of patients with hypertension. This, therefore, raises concerns about task shifting in the management of hypertension. In a mixed-methods study on task shifting in Ghana, quantitative results showed a significant improvement in knowledge following the intervention. Meanwhile, qualitative findings indicated increased confidence and proficiency among participants in managing hypertension (Gyamfi et al. 2017).

Hypertension education and all forms of health education, including medication adherence and lifestyle counselling, are often delivered by nurses (Rivera-Figueroa 2023). For nurses to adequately inform and empower the patient with knowledge and skills, they must be knowledgeable themselves. Self-management concepts such as physical activity, reduction in alcohol intake, weight management, healthy dietary choices, medication adherence and home BP monitoring are important components of hypertension management (Lobo et al. 2023). However, nurses’ knowledge of medication and lifestyle management of hypertension in this study was poor. The findings of this study provide valuable insights that will inform both the content and the implementation strategies of the guideline to be developed as part of the broader research project. Findings from Gyamfi et al. (2017) highlight the benefit of training nurses in hypertension management. It is also not surprising that Ojo et al. (2018), in their concluding findings, call for regular in-service training and certification in BP measurement.

Overall, this study observed that about half (57.1%) of nurses have average knowledge regarding hypertension, with a deficiency in some of these aspects. Different studies on nurses’ knowledge of hypertension corroborate the findings of this study (Al-Ahdal et al. 2024; Begum et al. 2023; Gyamfi et al. 2017; Ojo et al. 2018; Younis et al. 2024). In contrast, Begum et al. (2019) reported good knowledge of nurses in their study.

When comparing demographic data with knowledge levels, several variables were found to be significantly associated with overall knowledge: hospital of practice, age, nursing category, degree qualification, years of practice in the present unit and hypertension care in-service training. Age was associated with knowledge, with 63.6% (*n* = 7/11) of nurses aged 34 years and above falling within the good knowledge category, whereas younger nurses aged 23–33 years were more within the poor knowledge category (*n* = 70/103). This finding could be attributed to the likelihood that older nurses have accumulated more years of practice experience and thus greater exposure to continuous education. However, Myanganbayar et al. (2019) reported contrasting results, where age did not significantly influence knowledge. Similarly, in the present study, total years of nursing practice had no significant impact on knowledge, aligning with Myanganbayar et al. (2019) and Al-Ahdal et al. (2024). In contrast, duration of practice in the current unit that manages hypertension cases was associated with knowledge, with those having 1–5 years of experience in the present unit demonstrating either average or poor knowledge, and those with 6+ years having good to average knowledge. Based on these findings, it remains inconclusive to recommend targeted training exclusively for younger nurses, because overall years of experience did not consistently predict knowledge levels.

Most nurses in Ghana begin their training in nursing colleges, obtaining a Diploma in Registered General Nursing (RGN) and earning licensure after passing the Nursing and Midwifery Council (N&MC) examination. Others pursue a Bachelor of Science in Nursing at the university, also requiring N&MC licensure to practise as RGNs.

A third group, the enrolled nurses, complete certificate-level training and are licensed as Registered Nurse Assistants, a qualification below the diploma. Over time, both diploma and certificate-trained nurses often progress through top-up or bridging programmes to attain a bachelor’s degree, with the length of additional study depending on their prior qualification (Bell et al. 2013). Among nurses with good knowledge, the majority had a bachelor’s degree or higher (54.5%, *n* = 6). In contrast, nurses with lower qualifications accounted for the largest proportion of those with poor knowledge (60.2%, *n* = 62). This could be attributed to the fact that nurses who probably started at lower levels gained additional or refresher training on hypertension at their bridging programmes, an indication of the benefit of higher education in building theoretical knowledge. The studies of Younis et al. (2024) and Al-Ahdal et al. (2024) did not associate educational level with knowledge.

The essence of continuous professional development and refresher training in bridging knowledge gaps and sustaining clinical competence cannot be overemphasised. About 43% of nurses in this study reported post-school hypertension care training. This is higher than the 28.9% and about 30% in Ojo et al. (2018) and Machado et al. (2014) studies, respectively. Among nurses with poor knowledge, most (67.0%, *n* = 69) had not received additional training on hypertension care. In the average knowledge group, half had received training within the last 1–5 years (50.0%, *n* = 76). In a Pakistani study, 95.7% of nurses who received training over a period of 4 years at the time of participating in the study had poor knowledge, highlighting the need for continuous training on evolving guidelines (Younis et al. 2024).

The findings of this study highlight the crucial importance of nurses’ knowledge in the context of hypertension care, and most importantly, their role in self-management support. With hypertension being the number one risk factor for cardiovascular morbidity and mortality (Passi-Solar et al. 2020), particularly in LMICs, the implications of these findings on hypertension care in Ghana are vital.

### Strengths and limitations of the work

Our study comprehensively assessed nurses’ knowledge of hypertension care in the Northern Region of Ghana using validated tools to measure their current knowledge of ABPM, diagnosis and management. The findings will inform the content of the guidelines to be developed in the broader project.

Regardless of this study’s strengths, it is not without limitations. Because this study was conducted solely in the Northern Region, the findings may not be fully generalisable to the entire country. Also, the study conducted among urban nurses could be a limitation, raising concerns of selection bias. The relatively low Cronbach’s alpha for the knowledge test suggests minimal internal consistency, which may limit the instrument’s reliability. Therefore, the findings should be interpreted cautiously.

### Recommendations for further research

Further studies should go beyond knowledge to investigate how nurses’ knowledge is translated into actual practice, including ABPM and patient education on lifestyle modification and medication adherence. Another research direction is to conduct an interventional study by providing training on hypertension to assess knowledge acquisition and retention at different time points.

### Implications for policy and practice

The findings of this study, therefore, call for the design of a tailored continuous professional development programme on the prevention, diagnosis and management of hypertension for nurses. Hospitals and health institutions should institutionalise regular in-service training to ensure nurses’ knowledge is up to date with current hypertension guidelines. As part of the broader national non-communicable diseases (NCD) prevention and management strategies, the Ministry of Health, Ghana, should prioritise building nurses’ capacity to understand evolving hypertension guidelines and evidence-based practices.

## Conclusion

Overall, nurses working at TTH, TCH, and the TWH exhibited average to poor knowledge. Nurses have good knowledge of BP measurement; however, their knowledge of hypertension diagnosis, medication and lifestyle management was poor. High educational qualifications and in-service training are positively associated with knowledge. This underscores the need to prioritise education, training and ongoing capacity building for nurses in hypertension care. Prioritising the continuous development and capacity building of frontline healthcare providers will improve hypertension care, prevent cardiovascular complications and ultimately improve health outcomes.
